# The Use of Lean Six Sigma Methodology in the Reduction of Patient Length of Stay Following Anterior Cruciate Ligament Reconstruction Surgery

**DOI:** 10.3390/ijerph19031588

**Published:** 2022-01-30

**Authors:** Sinead Moffatt, Catherine Garry, Hannah McCann, Sean Paul Teeling, Marie Ward, Martin McNamara

**Affiliations:** 1 Beacon Hospital, Beacon Court, Bracken Rd, Sandyford Business Park, Sandyford, Dublin 18, D18 AK68 Dublin, Ireland; catherine.garry@beaconhospital.ie (C.G.); hannah.mc-cann@ucdconnect.ie (H.M.); 2 UCD Centre for Interdisciplinary Research, Education & Innovation in Health Systems, School of Nursing, Midwifery & Health Systems UCD Health Sciences Centre, D04 V1W8 Dublin, Ireland; sean.p.teeling@ucd.ie (S.P.T.); martin.mcnamara@ucd.ie (M.M.); 3 Centre for Person-Centred Practice Research Division of Nursing, School of Health Sciences, Queen Margaret University, Queen Margaret University Drive, Musselburgh EH21 6UU, UK; 4 Centre for Innovative Human Systems, School of Psychology, Trinity College, The University of Dublin, Dublin 2, D02 PN40 Dublin, Ireland; marie.ward@tcd.ie

**Keywords:** anterior cruciate ligament reconstruction, length of stay, non-value added, Lean Six Sigma, day case, surgery

## Abstract

**Background:** The purpose of this study was to reduce the length of stay of anterior cruciate ligament reconstruction patients within a private hospital in Ireland, reducing any non-value-added activity in the patient pathway, with the goal of increasing patient flow, bed capacity, and revenue generation within the hospital system, while maintaining patient satisfaction. **Methods:** We used a pre-/post-intervention design and Lean Six Sigma methods and tools to assess and improve the current process. **Results:** A reduction in inpatient length of stay by 57%, and a reduction in identified non-value-added activity by 88%, resulted in a new day-case surgery pathway for anterior cruciate ligament reconstruction patients. The pathway evidenced no re-admissions and demonstrated patient satisfaction. **Conclusion:** Six months post-project commencement, we had successfully achieved our goals of reducing our anterior cruciate ligament reconstruction patient’s length of stay. This study contributes to the growing body of published evidence which shows that adopting a Lean Six Sigma approach can be successfully employed to optimise care and surgical pathways in healthcare.

## 1. Introduction

### 1.1. Background

The study site for this intervention is a private hospital in Dublin, Ireland, providing acute care services in multiple disciplines, including orthopaedics, physiotherapy, and emergency medicine. Having opened in 2006, it currently has over 1300 staff, 210 beds, and eight operating rooms. At the commencement of our study, patients undergoing anterior cruciate ligament reconstruction (ACLR) surgery at the hospital had a two-day length of stay. This was in contrast with many other hospitals nationally and internationally, where patients undergo ACLR as a day-case procedure [1,2]. Within the peer-reviewed literature, there was also evidence that patients do not routinely require hospital admission post-ACLR surgery, as ACLR day-case procedures have demonstrated extremely low complication rates post-operatively [3,4,5]. In addition, the British Association of Day Surgery advises that 90% of patients who undergo ACLR should be discharged on the day of surgery [6]. With this in mind our study aims were as follows:

- To reduce the length of stay of ACLR patients by 80% within 9 months of this improvement project start date, thereby improving patient flow and bed occupancy in the hospital.
- To use an LSS approach to reduce any non-value added in the patient’s pathway, while ensuring that this had no negative impact on patient experience of care or post-operative outcomes.

The study site, under Irish healthcare regulations, operates independently of state health services and receives no state funding [7]. Care is funded through private health insurance. As the hospital receives no state funding, in order to remain operational, it must maximise revenue generation while providing high levels of quality patient care, patient satisfaction, and excellence in post-operative outcomes. Improving the turnaround times of elective surgery pathways, such as ACLR, can have a significant impact on revenue generation and improve patient flow without negatively impacting patient care.

Pre-intervention, the hospital performed 10 ACLR surgeries per month with an overnight stay for each of these surgeries, equating to 10 bed nights. On any given day in the month of November 2018, the hospital was operating at full capacity with no beds available for new admissions. Therefore, the main goal of this study was to investigate how, utilising Lean Six Sigma (LSS) methodology, we could reduce a patient’s length of stay to increase bed availability, improve patient flow, and increase revenue generation, while maintaining high levels of patient satisfaction and optimal post-operative outcomes. We aimed to achieve this reduction in length of stay through the identification of what is termed non-value-added activity. Non-value added is simply anything that does not add value to a process or system, in many cases in Lean ‘value’ is simply defined as the opposite to ‘waste’; ‘what is not waste is value’ [8].

The hospital site has a strong improvement culture, with 250 staff trained in LSS methodology by our academic partner university. The university programme has been instrumental in the development of LSS healthcare education and training nationally in Ireland [9], and hospital staff have been undertaking the programmes since 2017. Teams of interdisciplinary staff, educated and trained across all levels of LSS training and experience, from fundamentals training to degree level expertise, work across the hospital on specific projects and system-wide improvements. The hospital management team, therefore, saw the ACLR project as a proof-of-concept study for using LSS methods to review the process flow of an elective surgery type in a private hospital. An 80% reduction in length of stay was deemed an achievable goal post discussion with the relevant stakeholders, and 9 months an adequate timeframe for project completion.

### 1.2. Lean Six Sigma

Lean is a set of operating philosophies and methods that help create maximum value for patients by reducing waste and waits [10]. The approach has been developed from the automobile and manufacturing industries, and is a continuous process improvement system, comprising structured inventory management, waste reduction, and quality improvement techniques [11]. Lean utilises a continuous learning cycle that is driven by the ‘true’ experts in the processes of healthcare, i.e., the patients/families, healthcare providers, and support staff [12]. An important Lean strategy is to develop systematic approaches to defining value that integrate both the knowledge and clinical expertise of healthcare providers, and the patients’ preferences and needs [10].

Six Sigma involves seeking perfection through the elimination of variability [13]. While both Lean and Six Sigma have been used for many years, they were not integrated until the late 1990s and early 2000s [14] and, today, LSS is recognised as “a business strategy and methodology that increases process performance resulting in enhanced customer satisfaction and improved bottom-line results” [15]. LSS in healthcare has been identified as having an impact on health outcomes, process and quality of care, finance, and patient and staff satisfaction [16].

Lean Six Sigma is a powerful methodology that reduces waste and variation in an organisation, and ultimately minimises operating costs, optimises productivity, and maximises customer satisfaction [17]. LSS methodologies have been used successfully in a wide range of healthcare surgery pathways internationally [18,19,20]. A review by Mason et al. (2015) indicated a role for LSS quality improvement methodologies within surgery, with significant improvements demonstrated across a variety of outcomes within the pre-operative, operative, and inpatient settings [21]. Murphy et al. (2019) used LSS methodologies to increase the percentage of patients undergoing emergency hip fracture surgery within a 48 h timeframe from 55% to 85% at 6 months post-implementation [22]. Cima et al. (2011) showed that LSS can improve global operating room efficiency by decreasing pre-operative waiting times, improving on-time starts, decreasing turnover times, increasing daily operating room capacity, and gaining substantial support from the perioperative staff [23]. Brown et al. (2019) used LSS methodologies to improve their rate of day of surgery admission from 10 to 75% over a 19-month period, while reducing the duplication of perioperative tests from 83 to <2% [24]. LSS has also been shown to be effective in a paediatric hospital setting to decrease turnover and turnaround time [11]. Furter (2018) used LSS methodology to reduce the length of stay by 30% in 3 months in their emergency department, and increased patient satisfaction from 24% to 89.9% [25].

The hospital’s experience of utilising LSS for improvement, supported by the literature of its successful use in surgical pathways, was, therefore, a key determinant in our choice of methods. Our colleagues in the hospital have used LSS methodologies to achieve the completion of 100% of scheduled, elective orthopaedic surgeries within two working days of an outpatient appointment, as per their study goal [26]. Egan et al. (2021) used LSS methodologies to optimise the process involving the operating room nursing time assigned to prepare for surgeries and to maximise the nursing time available for patient care [27]. While O’Mahony et al. (2021) used LSS methodologies to redesign the supply chain to the operating room department to reduce associated costs and release nursing time to care [28]. The success of these projects that were undertaken in the hospital study site was achieved by utilising a combination of LSS methodologies and person-centred approaches to improvement, including respect for persons, gathering and listening to the voice of the customer, facilitating staff empowerment, and observing/understanding staff practices [29].

## 2. Methods

The objective of this study was to redesign and improve the process for the post-operative care of patients who have undergone ACLR surgery. We used LSS methodology due to its demonstrable impact on patient outcomes and patient experiences of care in healthcare settings, in both clinical and non-clinical areas [29,30], and its successful use within the study site previously [7,26,28,31,32]. To facilitate our improvement approach, we posed the question ‘Can the application of LSS to the process for ACLR post-operative surgery care reduce patient LOS?’ To answer this question, we used a pre- and post-intervention study design [33,34] that measures the occurrence of an outcome before and after a particular intervention is implemented. The approach of a pre- and post-intervention study involves the measurement of the variables of interest before and after the intervention in the same study site, on the assumption that any difference in measurement between ‘before’ and ‘after’ is due to the intervention [35], in this case, the intervention of an LSS improvement project. We recognised that this design has the limitation of ascribing with certainty results to an intervention [35]; however, the use of this design has been widely used to evaluate LSS in healthcare [22,24,28,36]. It was inherent in our study design that any outcomes from redesigning the process for post-operative care of ACLR patients could then be extrapolated and applied to all other surgeries within the specialty where relevant.

The Six Sigma Define, Measure, Analyse, Improve, and Control (DMAIC) framework structures improvement, and facilitates the identification of the root causes of problems and the formulation of control measures for any implemented solutions [37]. The DMAIC methodological approach was adopted in this study, and is widely described in the literature and in previous works of authors [38,39,40,41]. The individual but interdependent stages of DMAIC will each be expanded on throughout this paper to illustrate the methods used at each stage of the improvement. Specific LSS tools used throughout the project and our use of them within the DMAIC framework will be discussed in order of their use.

### 2.1. Project Team

As with other LSS project work within the study site [26,27,28], a project team was established to carry out the intervention. This team consisted of a chartered physiotherapist, a data analyst, and a project coordinator, all of whom undertook this study as part of their work toward the academic award of an LSS Green Belt. The chartered physiotherapist had knowledge of the process and the problem as they perceived it. The data analyst and project manager provided a fresh outlook on the process and problem, as they did not work in the area, and did not have a bias when approaching the problem. This was congruent with the study site’s approach to improvement, and its commitment to making LSS education and training accessible to team members from all disciplines and all levels of seniority [42].

As the core improvement team of three staff were working on the project, attending college, and held substantive posts, the team agreed to focus and pilot the improvement on one consultant’s surgical ACLR patients, from the consultant’s initial appointment to 30 days post-operative. The consultant chosen had the largest number of ACLR surgeries monthly. The age group of patients was between 16 and 50 years of age, as this is the main age group that sustains anterior cruciate ligament injuries. For the project, and in the interest of team building, we had the working name of ’Zero, the Ultimate Goal’. We now detail our methods using the DMAIC framework.

### 2.2. Define Phase

To define our project, we used the sandbox tool to establish exactly what was in and out of scope for this project [43]. We established, given the available time and resources, that the project scope would include all process steps, from the point where the patient visits the consultant in the clinic, to post-operative physiotherapy and discharge. The SMART assessment tool is used to manage the project goals, to determine if they are Specific, Measurable, Achievable, Relevant, and Timebound [35]. Each element is given a score out of 5, 1 meaning the goals would need significant improvement in each category, and 5 meaning that it meets the criteria. Our first goal (to reduce the length of stay of ACLR patients by 80% within 9 months of this improvement project start date, thereby improving patient flow and bed occupancy in the hospital) was given a score of 3 for specific, 4 for measurable, 3 for achievable, 4 for relevant, and 4 for timebound. Our second goal (to use an LSS approach to reduce any non-value added in the patient’s pathway while ensuring that this had no negative impact on patient experience of care or post-operative outcomes) was given a score of 3 for specific, 3 for measurable, 3 for achievable, 4 for relevant, and 4 for timebound. This was a useful tool, as it allowed us to really discuss and improve our goals.

Our specific goal focused our improvement project so that it was less general (focused on one procedure), while making sure that it was measurable. The further scoping of the project to one consultant meant that it was achievable and, as we have outlined, the relevance of the project was reflected in its aim to improve patient flow and bed occupancy in the hospital. Our goals were also timebound, allowing us to plan our improvement goals around a specific timeframe within which we would achieve our aims.

Based on our use of the SMART goals, it was determined that none of our goals would be hard to achieve or were unachievable. Factors that influenced the SMART review were the high support for improvement within the hospital, the support of an onsite academy linked to our academic partner, and the willingness of our pilot surgeon and the wider team to fully participate in each stage of the improvement.

We utilised the RACI tool, which is used to determine the roles and responsibilities that each relevant stakeholder will play during the project [44]. RACI is a method of communication planning to determine which stakeholders are Responsible (R) or Accountable (A) for each task, or whether they just need to be Consulted (C) or Informed (I) about the task, during each phase of the DMAIC project. This tool worked well with the stakeholder analysis tool, which was used to understand who the stakeholders were and where they currently stand on the issues associated with the project, and the power/influence tool, which determines the influence that the stakeholder has on the project [45].

The team took a person-centred approach to the improvement work, aware that person-centred care has an explicit focus on ensuring the client or patient is at the centre of care delivery [46], and that it is concerned with every person involved in the patient’s care, not just the patient [46]. Eliciting the voice of those involved in processes makes use of an LSS approach known as the voice of the customer, which seeks to articulate the voice of those involved [29] and to denote the expectations of the customer [47]. Seeking and understanding the staff voice was extremely important, as we needed to understand why the hospital ACLR patients were currently in overnight stay, and what measures we would need to implement to safely change this process to a day-case procedure with no detrimental impact on patient experience of care or care outcomes. The use of person-centred approaches in LSS projects has been shown to be effective [18,48] due to identified synergies with person-centredness [29]. This project also focused on gathering valuable feedback from the patient following their surgery, which was used to determine patient satisfaction, and to identify any issues that might have arisen during their stay. This invaluable insight, using the voice of the customer, helped us to fine-tune our pathway, taking into consideration both patient and staff comments and concerns.

**Figure 1:**
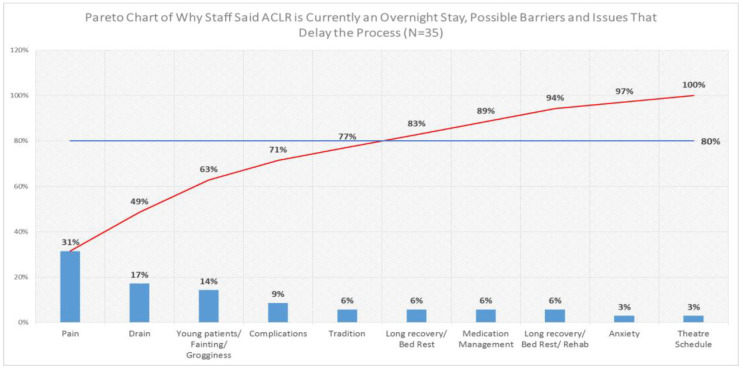
Pareto chart showing results from staff voice of the customer (VOC).

The voice of the customer data gathered from the staff was analysed using a Pareto chart (Figure 1), which showed that the staff involved in patient care felt that we needed to focus our attention on the patient’s pain, the length of time that the post-operative wound drain was in, the fact that they felt that we were dealing with a young cohort of patients that are more prone to higher levels of anxiety, fainting, and grogginess, possible complications that may arise, and the fact that traditionally, patients undergoing ACLR typically had a 2-day length of stay. These reasons accounted for 80% of the feedback collected and, therefore, were identified as the key areas for us to target for data collection. The staff feedback/voice of the customer indicated to us that a primary cause of concern was that any change in the current process from an overnight stay to a day case would require careful management of the patient’s post-operative pain relief.

This feedback identified the need to consider several pre-emptive measures, including establishing a pre-operative physiotherapy programme, ensuring the provision to the patient of user-friendly but comprehensive information regarding surgery, recovery times, the importance of their home exercise programme, and the nature of ACLR surgery as a day-case procedure. By giving the patient this necessary information pre-operatively, we aimed to reduce the patient’s anxiety and alleviate many of their concerns. Carli and Zavorsky (2005) highlighted the benefits of prehabilitation for a patient’s physical and psychological well-being, leading to the reduced potential for declines after surgery [49]. The pre-operative ACLR physiotherapy established for this project involved an individual appointment with the patient, and during this consultation, the physiotherapist would assess the patient’s knee and go through a series of exercises to improve knee range of motion, circulation, strength, and balance post-surgery, and give advice regarding wound management and the management of any knee swelling and pain post-operatively, and what to do if any complications occur. The physiotherapist would also go through how to use crutches (which is a walking aid) on the flat and on the stairs, as they must be used post-operatively by the patient. Ng et al. (2019) suggested that a dedicated perioperative pathway will have a significant impact on timely discharge [50]. Prehabilitation for orthopaedic patients has been evidenced to significantly improve pain, muscle strength (of specific muscle groups), and reduce the length of in-hospital patient stay [51]. Ditmyer et al. (2002) explained prehabilitation as a model that initiates the recovery process pre-operatively [52]. This model encourages pre-operative patient support to ensure that each patient functions to the best of their capability whilst waiting for their surgery. Ditmyer et al. (2002) also suggest that the prehabilitation of patients may result in better post-operative outcomes, such as improved independence and function, and reduced pain, as well as a better quality of life [52].

In addition to completing a staff voice of the customer, we completed a patient voice of the customer pre-intervention. Ten patients responded to a phone call, via which they were asked 9 questions. Here, 100% of the patients answered that they were extremely satisfied or satisfied with their ACLR surgery, and that they were well informed about their ACLR surgery and rehabilitation. Moreover, 100% reported that their pain was controlled on discharge, and 78% of patients did not experience any delays while they were inpatients. In addition, 50% said that they would be happy to have the procedure performed as a day-case procedure if they were required to have the surgery again, while 50% reported they would prefer to stay overnight. The main reasons given for the overnight stay were to ensure that their pain was controlled, that they experienced grogginess post-operatively, and that they would prefer to be monitored overnight in case of complications. Furthermore, 100% reported that their wound care was adequate post-operatively. When asked if they would change anything about the anterior cruciate ligament pathway to make it better, 33% of patients gave feedback, but only 1 was regarding the surgery itself. This feedback indicated that their post-operative physiotherapy differed slightly from their outpatient physiotherapy. When asked if they would benefit from pre-operative physiotherapy, 33% of patients said that they would benefit, 33% said they did not know, 11% said they would not benefit from it, and 22% did not answer. Many of the patients competed at a high level of sport, and received pre-operative rehabilitation prior to their surgery from their strength and conditioning coach or club physiotherapist, and this may have resulted in the mixed response to the last question. As per the staff’s voice of the customer, the patient voice of the customer also highlighted the significance of pain control and reducing anxiety by preparing the patient for surgery pre-operatively.

As stakeholders are critical to the success of LSS projects [53], we knew that our ability to mobilise commitment, via stakeholder engagement and effective communication, would be the difference between a successful project and a good idea that failed [54]. Therefore, following the results of the patient and staff voice of the customer, we liaised with the relevant key stakeholders, who included the orthopaedic consultant, anaesthetist, chartered physiotherapists, physiotherapy manager, operating room manager, orthopaedic ward managers, and the bed managers.

A key Six Sigma tool for identifying the ‘voice of the customer’ is the critical to quality tree. This is a tool that is used to:

1. Identify the needs of the customer (e.g., patients, staff, and family).
2. Identify what drivers the organisation should have in place to meet these needs.
3. Identify the metrics to ensure that this driver is meeting the need.

**Figure 2:**
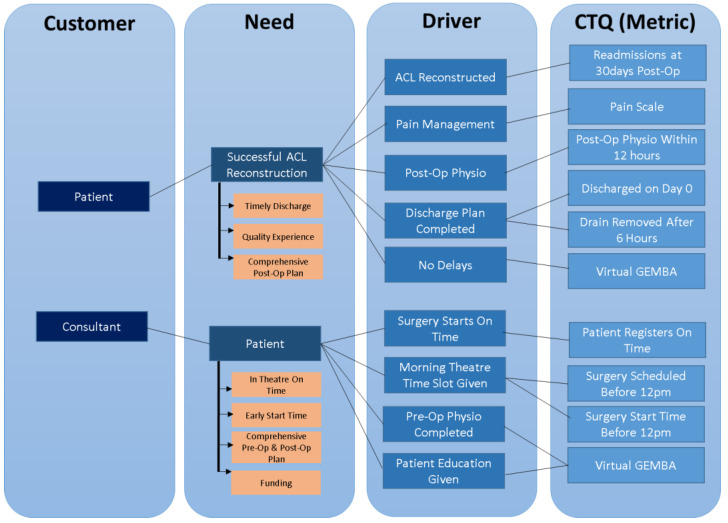
Critical to quality tool.

The critical to quality tree takes information collected from customers and translates it into critical and specific process requirements that are measurable [55]. Lean, Six Sigma, and LSS all seek to define what exactly is valuable in a healthcare setting from the perspective of the customer or end-user [56,57,58,59]. Our use of the critical to quality tool (Figure 2) was also extremely important in the Define phase, as it allowed us to understand what we had to analyse and how to best collect that data. Our critical to quality tool enabled us to figure out what we needed to measure to find out the improvements we needed to make to meet our goals, and ultimately successfully change the ALCR pathway to a day-case procedure.

**Figure 3:**
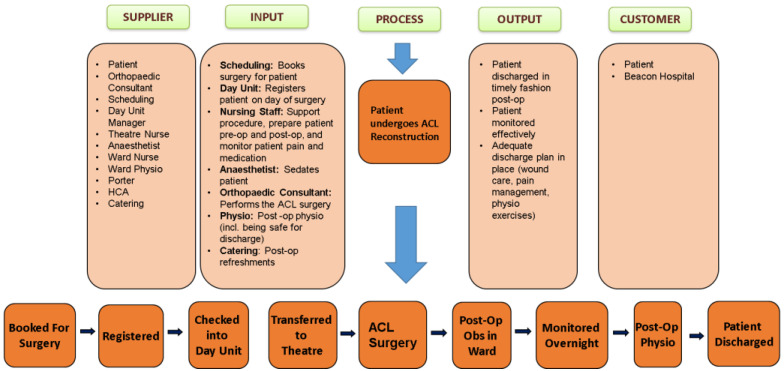
SIPOC tool.

To facilitate the cohesiveness of the team, consolidate our aims, and make our project objectives clear, we utilised an LSS tool called SIPOC (Suppliers, Inputs, Process, Outputs, and Customers), illustrated in Figure 3. Our use of SIPOC also enabled us to become more familiar with the current process of ACLR surgery and patient flow.

SIPOC is used to identify how some input is transformed into a value/outcome [55]. SIPOC is based on customer satisfaction, wherein in our case, the customer is both the patient and the hospital itself. Both customers are a beneficiary of the process in the end. In our process, there are several suppliers, including the consultant, nursing staff, physiotherapy team, and schedule. The input section identifies the role that the suppliers play within the process, while the output is the desired outcome following the process, resulting in a satisfied customer. Using the SIPOC tool identified that, within our process, the patient is both a supplier and a customer, as they are involved in the whole process from beginning to end.

### 2.3. Measure Phase

Initial Pre-intervention/Baseline Data Collection.

**Figure 4:**
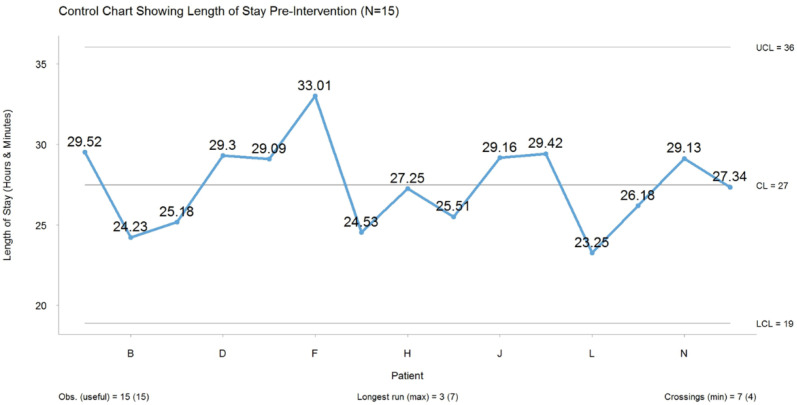
Length of stay of ACLR patient’s pre-intervention. CL = Centre Line, UCL = Upper Control Limit, LCL = Lower Control Limit.

Our baseline data indicated that the median length of stay for ACLR patients (*n* = 15) before the intervention started was 27 h (Figure 4). Audit data enabled us to understand the non-value-added and value-added composition of this 27 h period, and it was found that 15 h (60%) was non-value added to the process, while 11 h (40%) was value added.

During the measure phase, we made use of process mapping, which is used to visualise a process at a more detailed level than SIPOC, and makes the various steps in a process obvious through visual mapping [60]. We developed an ‘As Is’ Process map (the process state pre-intervention, often referred to as current state) to understand the length of the process, from when the patient is booked for surgery until their discharge from the hospital. This can also give us an indication of where the non-value-added actions may lie. Our data collection plan focused on key metrics, which were identified from our SIPOC as:

- Length of stay from when the patient arrives in the day unit until they are discharged home.
- Number of anterior cruciate ligament procedures performed from April 2019 to October 2019.
- Hospital occupancy, measured monthly from project start in December 2018.
- Pain score, measured hourly.
- Length of time the drain is in situ.
- Thirty-day re-admissions.
- Any delays that occurred were documented.
- Time of post-operative physiotherapy.

To validate our understanding of the process ‘As Is’, we developed an audit tool to be completed locally by orthopaedic nursing staff to facilitate our data collection. This data collection contained information on the above key metrics, and included the time of patient’s arrival onto the ward, the pain score on arrival, the amount in the patient’s drain (a drain is a plastic tube that is inserted into the patient wound, and it is used to collect fluid post-operatively to decrease the theoretical risk of intraarticular adhesions and joint stiffness) [61], hourly pain levels, the time of drain removal, and the time of post-operative physiotherapy.

The time of post-operative physiotherapy is important, as physiotherapy will only take place if the patient’s pain is controlled, and after the drain is removed, to facilitate mobilisation and thereby discharge. This accuracy of the staff audits was validated when we compared the data to our hospital-wide electronic patient record (EPR). This system records the patient’s arrival time onto the ward, the time of physiotherapy assessment, pain scores, the time of drain removal and amount of fluid collected, and discharge times.

### 2.4. Analyse Phase

During the analysis phase, we collated all our collected data in Excel and used R Statistical Software for graphing and analysis. We determined that the average length of stay for patients (*n* = 15) was 27 h from registration to discharge, as depicted in Figure 4. A value added and non-value added illustration pre-improvement assisted us in where exactly to improve. We found that the majority of the non-value added was in the overnight stay (it accounted for 54% of the patient’s time in the hospital). The data from our audits and process maps were analysed using a modified fishbone diagram to carry out a root cause analysis on the non-value added identified from these methods [11].

This highlighted several issues with the current process, which included concerns about patients’ post-operative pain, how long the surgical wound drain was in place, operating room utilisation, operating room schedule, and other concerns about how, traditionally, this was an overnight procedure. We found that the fishbone helped us to identify areas for improvement, which we split into categories according to whether tasks could be performed either rapidly, with further planning, or at a later stage; effectively, we triaged our root cause analysis findings [62]. For example, ACLR patients were currently staying overnight due to reported pain levels being significant. Our collated data, when mapped to the fishbone diagram, highlighted that patient pain, the surgical drain, operating room utilisation, operating room schedule, and ’we’ve always done it this way’ or ‘tradition’ were all root causes as to why the ACLR surgery currently was performed as an overnight procedure.

### 2.5. Improve Phase

In the Improve phase of DMAIC, we built on our analysis for the fishbone diagram, and brainstormed solutions with our stakeholders/participants to facilitate the development of an implementation plan to actualise the improvements. The practicality of solutions was discussed at length with our multidisciplinary team of stakeholders, which was extremely useful, as it allowed us to conceptualise solutions and understand how useful they would be. For example, a solution could have been to have the patient recover in the patient lounge post-surgery. However, when we investigated how practical this solution was, we discovered that this was not feasible, as the patient’s knee must be elevated post-operatively, and the chairs in this area were not suited for this type of recovery.

We decided to pilot our solutions on 1 patient, which would allow us to identify any issues that might arise and correct them before rolling them out to all other patients. The co-designed solutions that we focused on were to reduce the length of time the drain was in situ, to change the anaesthetic and pain medication to better suit day-case surgery and facilitate earlier discharge, to complete post-operative physiotherapy and education, to recover on the ward, and to book the patient into an early slot for theatre (before 12 p.m.). Lucocq et al. (2021) reported that patients positioned last on the theatre list in their study were more than 5 times more likely to be admitted post-ACLR surgery, as a late arrival time onto the ward allocates less time post-operatively to plan for a safe discharge [4]. The pilot commenced once the consultant booked the patient in for surgery, and was completed once they were 30 days post-discharge.

Our ten-step implementation plan, as outlined below, was established to facilitate our first-day case ACLR surgery.

1. Inform stakeholders of pre-intervention ‘As Is’ situation.
2. Discuss with the operating room scheduling team (this is the team that schedules the patient’s slot in theatre after the consultant has booked them in for the surgery) having ACLR patients first on the operative list for surgery.
3. Pre-operative physiotherapy to be initiated and consultant to inform the patient of day-case status.
4. Patient to be booked by the consultant as normal, but, in this instance, is scheduled as a day-case procedure (as opposed to an overnight stay, provided that the patient meets all inclusion criteria mentioned above).
5. Verify with scheduling and billing about the change of patient status from an inpatient to a day-case procedure.
6. Liaise with ward managers and bed managers as to recovery on the ward as opposed to the day unit.
7. Reminder emails with audit attached to be sent to all relevant stakeholders 2 days pre-surgery.
8. LSS team rota to be established for the day of surgery to assist staff with data collection.
9. Collect the audit form on the morning after discharge, and discuss any issues that may have arisen with the ward manager.
10. Analyse audit data.

The above ten steps illustrate what seems like a simple pilot, but which involved intensive stakeholder engagement and discussions which were time-consuming. However, the time put into this engagement and the project overall was seen as having its return on investment for the organisation in anticipated results.

Interventions were put in place prior to the first day-case/pilot patient, as outlined in our implementation plan above. These included pre-operative physiotherapy and education. The patient was also to be put first on the operating room schedule on that day to facilitate same-day discharge. The consultant, as mentioned, provided the patient with information regarding their surgery and booked the patient in. The patient had their post-operative recovery on the orthopaedic ward as opposed to the day unit. This recovery location was for two reasons: firstly, the day unit was already functioning at full capacity, so did not have space for more day-case procedures; and secondly, by recovering on the orthopaedic ward, in the event of a patient having a post-operative complication, they could remain in the same bed overnight. Should no complications occur, they could be discharged, allowing another patient to take their bed on the ward, should it be required. As mentioned, this improved patient flow throughout the hospital.

## 3. Results

The first day-case/pilot was performed on a patient requiring ACLR and a meniscectomy. A meniscectomy is the surgical removal of the meniscus (which is a piece of cartilage/connective tissue that provides a cushion between the thigh and shin bones). The meniscus is frequently damaged when a patient tears their anterior cruciate ligament. The patient arrived at the hospital at 06:03 a.m. and was scheduled for the operating room at 07:30 a.m. The patient was returned to the ward to recover at 11:00 a.m., where they recovered until the drain was removed at 18:00 p.m., and physio was completed at 18:40 p.m., prior to discharge at 20:32 p.m. On a scale of 0 to 10, with 0 being no pain and 10 being unbearable pain, the patient indicated their pain level to be 3 out of 10 on discharge; therefore, the patient was deemed fit to return home, following a stay of 14.5 h. A follow-up phone call was carried out the following day and no issues or concerns were voiced by the patient.

We were able to show a 13 h reduction in length of stay when patients were treated as day cases rather than inpatients. The first post-intervention patient stayed in the hospital for 14.5 h, after being admitted at 06:03 a.m. and discharged the same day at 20:32 p.m.

Following the post-intervention pilot, a further 17 patients were to be included in our analysis over a 6-month period (April–October 2019). Each patient met our criteria for inclusion in this study, and each was followed up with a phone call post-surgery and monitored for any 30-day readmission. We compared the core datasets, including data on length of stay and pain score, to those of the 15 patients pre-intervention. The length of stay data were determined to have a normal distribution using the Shapiro–Wilk normality test for the data pre-intervention (*p* = 0.58) and post-intervention (*p* = 0.22). The median length of stay pre-intervention was 27.6 h (SD = 2.61) (*n* = 15), while in our post-intervention group, the median LOS was reduced to 11.7 h (SD = 1.66), which showed a decrease of 15.9 h. This decrease was deemed statistically significant using the Welch Two Sample *t*-Test (*p* < 0.001). When monitoring for pain (*n* = 21), it was shown that the pain scores post-surgery, on return to the ward, was an average of 4 pre-intervention (SD = 2.36) but was 0 post-intervention (SD = 1.96). On discharge, the average pain score was 2.5 pre-intervention (SD = 1.41) and was 3 post-intervention (SD = 2.06). Although the pain score was shown to be slightly higher post-intervention, this was not deemed to be statistically significant (*p* > 0.5). Along with measuring pain, we also tracked the length of time that the drain remained in situ post-operatively. Pre-intervention, the median length of time was 21.7 h (SD = 2.79), which represented the overnight stay of the patient. Post-operatively, this was reduced to a median of 7.3 h (SD = 1.29) (*p* < 0.001). In the comparison of our value added analysis before and after our interventions, we were able to show an increase in value-added time from 40% to 93%, which is a 132.5% increase in our VA time. By comparison, we had also shown an 88% decrease in non-value-added time, from 60% down to only 7%. Table 1 outlines the results of all statistical tests for the length of stay, pain score, and length of time the drain was in situ.

Follow-up phone calls were conducted for 22 patients, post-operatively, who were each asked nine questions (as per the patient voice of the customer pre-intervention) regarding their satisfaction with the procedure being conducted as a day case. Of these, 11 patients answered, and the remaining patients did not answer. If they did not answer a voicemail was left on their phone, if possible. Of the 11 patients that did answer, 100% of them were either satisfied or extremely satisfied with the overall treatment that they received. All these patients reported that their wound care information and post-operative physiotherapy were comprehensive and had no suggestions for improving the ACLR patient pathway. In addition, 100% reported that they experienced no delays while they were in the hospital. When asked if they were to have this surgery again, would they be happy to have it conducted as a day case, only one patient said that they would prefer an overnight stay ‘in case they experienced complications’, even though they did not post-operatively.

### Control Phase

Once the process had been improved, we then issued our control plan to all stakeholders to ensure that the process continued. Our control plan included six process steps, which were to be continued by five different departments, monthly. The scheduling department was responsible for booking the patient in as a day-case procedure and to recover on the ward, the theatre scheduling team were to ensure that the ACLR patients were booked into early theatre slots, the orthopaedic ward manager was to monitor the length of stay, the physiotherapy department was to complete pre-operative physiotherapy and education and to complete follow-up phone calls to assess patient progress/ensure that there were no post-operative complications, and the quality team monitored any unplanned 30-day readmissions. Throughout the project, we contacted the ward staff and stakeholders to see if any issues were arising. Any issues that were reported were dealt with. There were no significant barriers met or issues that could not be resolved quickly by the implementation team.

## 4. Discussion

Anterior cruciate ligament injuries most often occur in athletes participating in sports that involve cutting, pivoting, jumping, or landing [63], such as football, basketball, and downhill skiing. For this young cohort of patients, it may be their first experience of hospital or surgery, and recovery from the same, and this may result in increased levels of pain and anxiety. Traditionally, in the study site, these patients have been admitted as inpatients and kept for observation overnight, before being discharged the following morning. Lutz et al. demonstrated that a similar pain intensity after primary arthroscopic ACLR was experienced as an outpatient and as an inpatient [5]. Therefore, they reported that there is no medical need for ACLR patients to be kept overnight once the pain is controlled and there are no post-op complications [5].

This project aimed to reduce the length of stay of ACLR patients, from an overnight stay to a day case, in a safe manner, with the aim of freeing up more beds for a hospital that is running at full capacity daily. This would also allow the patients to return to the comfort of their own home, post-surgery, for recovery. Many authors have shown that patients undergoing ACLR as day-case procedures have reported higher overall satisfaction than inpatients due to reduced admission time, prompt discharge to a familiar home environment, and the ability to self-administer analgesia in a timely manner [64,65,66].

The first step in achieving this goal was to implement pre-operative education and physiotherapy. This intervention was based on the voice of the customer feedback from the staff, who raised concerns about patients being mentally prepared, with 80% of those asked suggesting that per-operative education would help alleviate patient anxiety. We also implemented follow-up calls post-operatively, while the patient was recovering at home, to understand their view of the process, and identify any issues that may have arisen. This vital feedback from the patient allowed us to determine that all patients had been satisfied with their surgery and their length of stay in the hospital. Another metric of success was to monitor the 30-day readmissions for these patients, of which none were re-admitted. Khan et al. showed that 80% of patients who had followed a detailed clinical pathway with standard operating procedures during ACLR were discharged the evening of their admission, whereas this rate was only 16% in the “standard” group of patients, who had no specific organisation [67]. As Lefevre et al. (2014) noted, the protocolisation of a clinical pathway is the cornerstone of treatment organisation [68].

To measure the success of this project, we also compared the results from our baseline to post-intervention, with the aim of reducing the length of stay from a median of 27.6 h, while maintaining a low pain score for the patient. Following the implementation of our interventions, the median length of stay was reduced by nearly 16 h, while maintaining pain on discharge at a low score of 3 out of 10. Both metrics showed that we met our goals. This reduction was clearly shown in the reduction of the non-value added in our process from 60% to 7%. This 53% reduction in non-value added represented the length of time spent by the patient recovering/sleeping overnight, with the drain in situ.

This successful implementation was due to a multitude of factors, particularly due to successful stakeholder buy-in. As mentioned earlier, there is a strong LSS culture in the hospital, which greatly enabled all stages of our project. In addition, at all steps in this process, we engaged with the relevant stakeholders to discuss barriers to success and possible ways to improve the current system, with each step being signed off before implementation. Meetings were held with the operating room scheduling team to enable the booking of ACLR patients first on the list, while discussions with the pain team allowed for a better understanding of current pain relief given to these patients, and what would be the best choice for the day-case procedures. Following these meetings, we liaised with the consultant surgeon and anaesthetist to discuss our findings and determine the best course of action going forward. These discussions included the reduction in the length of time for which the drain will be in situ, and the use of tranexamic acid and other types of pain relief best suited to day-case procedures, with the goal of optimal pain relief and recovery for our cohort of patients. Johns et al. (2021) concluded from their study that the use of tranexamic acid in ACLR surgery results in reduced joint drain output and hemarthrosis, and improved pain scores and range of motion in the initial post-operative period, without complications or thromboembolic events [69]. The changes that were introduced because of these discussions led to earlier mobilisation and physiotherapy while ensuring pain was controlled, patient satisfaction was maintained, and ultimately earlier discharge times. Khan et al. (2012) highlighted the importance of the close coloration between the surgeon, anaesthesiologist, and the department’s lead nurse [67].

We have now discussed the success of this project in reducing the length of stay for the patients, and the factors that enabled us to reach that target. By reducing the length of stay of our ACLR patients we thereby increased bed capacity in the hospital. This project was focused on the patients of one consultant. By analysing their data alone, we have shown that the reduction in the length of stay, by nearly 16 h, resulted in the ability to free up 17 beds for other patients who needed to be admitted overnight, in a 6-month period. This reduction also resulted in a saving of 11.3 bed days for the hospital, while also having a significant cost reduction for the patient of EUR 950 (overnight rate).

**Figure 5:**
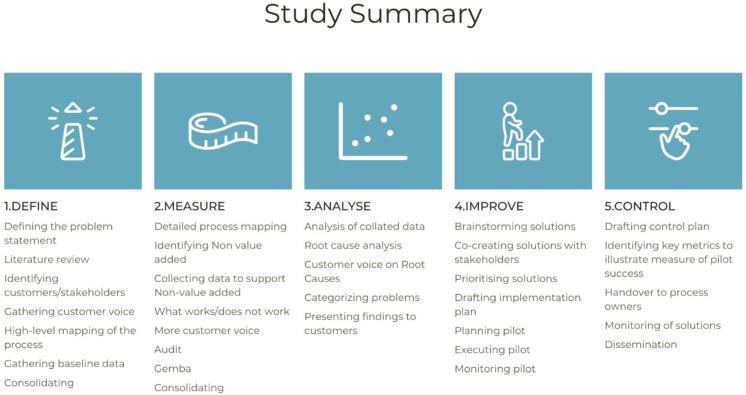
A summary of our study using the DMAIC framework.

In summary, we used the DMAIC framework to guide our work. In the Define phase, we utilised a multitude of LSS tools (SMART tool, RACI, voice of the customer, critical to quality tool, and the SIPOC tool) to firmly define the parameters of our project, consolidate our aims, and clearly understand the current anterior cruciate ligament pathway. The measurement of data took place over a 6-month period, and we then analysed the data, which enabled us to develop improvements. Once implemented, we ensured that a robust control plan was in place. An overview summary of our study is displayed in Figure 5.

### 4.1. Implications

Following the successful implementation of this project with this one consultant, the hospital is incrementally introducing this new process to all consultants that perform ACLR surgeries to further increase the number of available beds for the hospital, while reducing costs and the length of stay for patients. This is dependent on successful engagement with other consultants who perform this surgery, but this project has shown the benefit to both the patient and the hospital, which has facilitated consultant interest and engagement. The LSS process used in this project, our person-centred approach to key engagement with our stakeholders, and our incremental approach to system-wide change could be adapted to other elective orthopaedic surgeries, both within the hospital and within other surgical centres.

### 4.2. Limitations

Patients from only one consultant were included in this six-month project; therefore, patient numbers were low. As mentioned, only one consultant was selected, and this was mainly due to time constraints on the team members, who were attending college, completing this project, and performing substantive posts simultaneously. As this intervention is extending to all orthopaedic consultants in the hospital who perform ACLR surgeries, other unidentified factors may arise. One such issue may be the lack of early morning theatre slots. We identified that in order for our ACLR’s to be completed as day-case procedures, we require an early theatre slot. As this project is rolled out to all orthopaedic consultants performing ACLR, it may mean that some patients will be scheduled later than 12 p.m. (this was the time that was identified as the cut-off point for which day-case ACLR procedures can be performed, and after this time ACLR surgeries resulted in overnight stays). Should this issue arise, our team will have to use LSS to facilitate root cause analysis and identify solutions.

Although our team worked well together, we recognise that a limitation may have been the absence of a direct ‘process owner’, an individual directly involved in the process. Ideally, this would have been the orthopaedic consultant who agreed to pilot the ALCR improvement or one of his team. Unfortunately, as they worked across multiple hospital sites, they could not commit time to become part of the actual project team. This may have impacted the intervention, as their visible leadership and expertise, a key component of LSS interventions [30], might have impacted the results and how they were interpreted. The presence of process owners may also have increased the credibility of the intervention, with a more appropriate team composition and physician involvement being a critical input that influences key objectives, including leadership and reflexivity, which in turn impact outcomes [70]. However, the consultant and his team were key stakeholders who worked with the project team with a sense of shared purpose. Shared purpose results when a group of individuals aligns their belief systems or values with a common challenge, vision, or goal—in this case, the ALCR improvement—and has been shown to unify diverse groups in collaborative activities, enabling participants to work together creatively in the same direction [71]. We were fortunate in that we had good stakeholder engagement and a shared purpose from the beginning to the end of the study.

## 5. Conclusions

This project has been successful in reducing ACLR patients’ length of stay, and significantly reducing non-value added from the patients’ pathway. Six months post-project start, we achieved a 57% reduction in ACLR patients’ length of stay post-intervention, thereby increasing patient flow, patient satisfaction, bed capacity, and revenue generation. We reduced our non-value added from 60% to 7%; an 88% reduction from the project start. A significant contribution to the success of the project was the involvement of stakeholders and members of the multidisciplinary team from multiple departments throughout the hospital. The team approach resulted in the ACLR surgeries being performed as a day-case procedure, as opposed to an overnight stay, while maintaining high levels of patient care and satisfaction, as demonstrated by the results of patient questionnaires, the follow-up phone calls, and zero readmission rates. Using LSS methodologies, we clearly identified where the non-value added lay in the patient’s pathway while they were an inpatient. By executing our 10-step implementation plan, we achieved our overall goal, resulting in day-case ACLR surgeries. The success of this project will hopefully result in the roll-out of these methods to other elective surgery pathways. These process improvement techniques improved quality, while also reducing operating costs and inventories, which translated into significant savings for the hospital. This resulted in a win–win situation for all [72]. This paper contributes to the growing body of published evidence that LSS methods can be successfully employed to optimise care delivery in surgical pathways in healthcare. Additionally, this pilot project demonstrates that LSS methodologies can be seen as more than what McNamara and Teeling (2019) refer to as a decontextualised toolkit, and emphasises its use for engaging with all of those involved in the complex processes inherent in healthcare across the entire health system [9]. We contend that this paper demonstrates that applying LSS and person-centred methodologies to process improvement synergistically can yield results for all stakeholders at the level of patient, staff, and organisation.

## Figures and Tables

**Table 1:** Table indicating the result of statistical tests on all data (*** *p* < 0.001).

Statistical Test	Pre-Intervention	Post-Intervention
**Average Length of Stay**		
Shapiro–Wilk Normality Test	0.6	0.2
Mean LOS	27.7	12.3
Median LOS	27.6	11.7
SD LOS	2.6	1.7
Welch Two Sample *t*-Test	6.197 × 10^−16^ ***	
**Average Pain Score**		
Mean Pain After Surgery	4	0
SD Pain After Surgery	2.4	1.9
Mean Pain At discharge	2.5	3
SD Pain At Discharge	1.4	2.1
Welch Two Sample *t*-Test (after surgery)	0.18	
Welch Two Sample *t*-Test (at discharge)	0.65	
**Average Length of Time Drain In Situ**		
Mean Drain	21.9	7.4
Median Drain	21.7	7.3
SD Drain	2.8	1.3
Welch Two Sample *t*-Test	1.062 × 10^−6^ ***	

## Data Availability

The data from this study is available in and presented as part of this paper.
